# Calcium scoring using virtual non-contrast images from a dual-layer spectral detector CT: comparison to true non-contrast data and evaluation of proportionality factor in a large patient collective

**DOI:** 10.1007/s00330-020-07677-w

**Published:** 2021-01-20

**Authors:** Felix G. Gassert, Claudio E. Schacky, Christina Müller-Leisse, Florian T. Gassert, Gregor Pahn, Karl-Ludwig Laugwitz, Marcus R. Makowski, Jonathan Nadjiri

**Affiliations:** 1 Department of Diagnostic and Interventional Radiology, School of Medicine & Klinikum rechts der Isar, Technical University of Munich, Ismaningerstr. 22, 81675 Munich, Germany; 2 Philips CT Clinical Science, Hamburg, Germany; 3 Department of Internal Medicine I, School of Medicine & Klinikum rechts der Isar, Technical University of Munich, Munich, Germany

**Keywords:** Coronary artery disease, Vascular calcification, Computed tomography angiography

## Abstract

**Objective:**

Determination of coronary artery calcium scoring (CACS) in non-contrast computed tomography (CT) images has been shown to be an important prognostic factor in coronary artery disease (CAD). The objective of this study was to evaluate the accuracy of CACS from virtual non-contrast (VNC) imaging generated from spectral data in comparison to standard (true) non-contrast (TNC) imaging in a representative patient cohort with clinically approved software.

**Methods:**

One hundred three patients referred to coronary CTA with suspicion of CAD were investigated on a dual-layer spectral detector CT (SDCT) scanner. CACS was calculated from both TNC and VNC images by software certified for medical use. Patients with a CACS of 0 were excluded from analysis.

**Results:**

The mean age of the study population was 61 ± 11 years with 48 male patients (67%). Inter-quartile range of clinical CACS was 22–282. Correlation of measured CACS from true- and VNC images was high (0.95); *p* < 0.001. The slope was 3.83, indicating an underestimation of VNC CACS compared to TNC CACS by that factor. Visual analysis of the Bland-Altman plot of CACS showed good accordance with both methods after correction of VNC CACS by the abovementioned factor.

**Conclusions:**

In clinical diagnostics of CAD, the determination of CACS is feasible using VNC images generated from spectral data obtained on a dual-layer spectral detector CT. When multiplied by a correction factor, results were in good agreement with the standard technique. This could enable radiation dose reductions by obviating the need for native scans typically used for CACS.

**Key Points:**

• *Calcium scoring is feasible from contrast-enhanced CT images using a dual-layer spectral detector CT scanner.*

• *When multiplied by a correction factor, calcium scoring from virtual non-contrast images shows good agreement with the standard technique.*

• *Omitting native scans for calcium scoring could enable radiation dose reduction.*

## Introduction

Ischemic heart disease is the most common global cause of death, with high mortality reported in the developed countries [1]. Therefore, early detection of coronary artery disease (CAD) is crucial for the prevention of adverse events through dedicated therapy. Hence, non-invasive cardiac imaging by computed tomography plays an increasingly significant role in diagnosis of CAD [2–4]. Besides contrast-enhanced imaging for the evaluation of vascular stenosis, several studies showed that the determination of coronary artery calcium scoring (CACS) is an essential prognostic factor and a strong and independent predictor of cardiovascular events, such as myocardial infarction and sudden cardiac death [3, 5, 6]. The most commonly used method to evaluate patients’ burden of CAD is the Agatston score, which measures the amount of calcium present in each lesion scaled by an attenuation factor and summed over all lesions [7].

Whereas currently both contrast-enhanced CT imaging for stenosis determination and non-contrast images for CACS are widely established for the diagnosis of CAD, several methods using spectral imaging including the possibility to generate virtual non-contrast (VNC) images for calculation of CACS have previously been proposed in multiple studies [8–10]. Dual-energy imaging is based on simultaneous acquisition of two CT datasets at different x-ray spectra—either by data acquisition at two different x-ray tube voltages (dual-source CT, kV switching) or by energy separation in the detector (dual-layer CT). Based on decomposition into two base materials (soft tissue and iodine), a virtual non-contrast image can be generated from a contrast-enhanced image [11, 12]. Especially the novel technique of spectral CT imaging using a dual-layer spectral detector CT (SDCT) system might help to overcome some limitations of other dual-energy techniques. For example, there is no temporal or projection offset using the dual-layer method. Furthermore, the dual-layer method has been reported to be a reliable method of dual-energy imaging [13, 14].

In a preliminary study, Nadjiri et al showed that the use of spectral imaging with SDCT for the determination of CACS from contrast-enhanced coronary computed tomography angiography (coronary CTA) may be a feasible alternative and reaches good agreement with the conventional technique. Nevertheless, the results of CACS (Agatston score) from VNC images needed to be multiplied by a proportionality factor of 1.83 to match the results from TNC images due to underestimation of plaque density and plaque volume with the VNC data. Furthermore, the sample size was small and patients without coronary calcifications were included in statistical analysis, resulting in a higher correlation [5].

The use of VNC images generated from spectral data could reduce radiation exposure by omitting additional native scans when performing a CTA. For establishing this method in everyday clinical practice, we sought to evaluate a more representative patient cohort and compare VNC with true non-contrast imaging. Furthermore, this study analyzes the extent and causes of needed proportionality factors.

## Material and methods

### Study population

Approval of the Institutional Review Board had been obtained prior to this study. Written informed consent was waived for this retrospective analysis of routinely acquired imaging and clinical data. All patients who underwent CACS and coronary CTA due to suspected CAD using spectral CT at our institution were eligible for the study. All patients without calcified plaques in true non-contrast (TNC) images (corresponding to a calcium scoring of 0) were excluded from statistical analysis.

### Computed tomography scans

Heart rates lower than 60 bpm were preferred for all examinations. Therefore, in patients with heart rates above 60 bpm, we administered up to 20 mg metoprolol intravenously before scanning. Additionally, 0.8 mg nitroglycerin was administered just before scanning for vasodilatation of coronary arteries when systolic blood pressure was above 100 mmHg.

A 64-slice single-source dual-layer spectral CT system was used for imaging (IQon; Philips Healthcare). The firmware used on the scanner was Version 4.7.0. A tube voltage of 120 kVp was applied for both native and contrast-enhanced scans. Tube current time product for CACS was 35 mAs and 140 mAs for coronary CTA. Reconstruction of native scans was performed with a XCB kernel and reconstructions of coronary with CB (both standard cardiac kernels). Both reconstructions had identical spatial resolution and slice thickness.

ECG-triggered sequential scans were used for the acquisition of non-contrast images (as reference) and contrast-enhanced images. Bolus tracking was applied for the timing of the contrast phase in contrast-enhanced images. In total, 80 ml of contrast agent (Ultravist 370, Bayer, Bayer AG, iodine content 370 mg/ml) was used for the contrast-enhanced scans, applying a flow rate of 4–6 ml/s followed by a 50-ml bolus of saline chaser.

### Post processing

VNC images were generated from contrast-enhanced images using a software package which is certified for medical use and commercially available (IntelliSpace Portal version 10, Philips Healthcare). A slice thickness of 2 mm was used for both TNC and VNC images.

Calcium score from both TNC and VNC was determined (IntelliSpace Portal). The threshold for plaque inclusion was set to 130 Hounsfield units (HU) as previously described in the literature and clinically used [7, 13, 15, 16]. All intraluminal (coronary arteries) plaques were selected and used for calcium scoring. Results using TNC and VNC data were compared.

### Statistical analysis

All statistical analyses were performed using the statistical package R version 3.2.4 (R Foundation for Statistical Computing). Categorical variables are expressed as frequencies and percentages, continuous variables are expressed as mean ± standard deviation. For analysis of method agreement, Bland-Altman plots were applied. Method of least squares was used for linear regression between methods. Pearson’s correlation coefficient was calculated for measuring the association between variables. A *p* value < 0.05 was considered statistically significant. G*Power was used for the analysis of post hoc study power.

## Results

### Study population and radiation dose

One hundred three consecutive patients with suspected CAD were scanned using the dual-layer SDCT system between January 2018 and March 2020. Fifty-five patients (53.4 %) were referred to coronary CTA for atypical cardiac symptoms, 26 patients (25.2 %) for typical cardiac symptoms, and 22 (21.4 %) for evaluation of operability. Out of the study population, 32 patients (31.07 %) were diagnosed with obstructive coronary artery disease. Thirty-one patients were excluded from primary statistical analysis due to calcium scoring of 0 as determined by the TNC images. Twenty-three of these patients had a CACS of 0, three of these patients had a CACS of 1, four had a CACS of 2, and one had a CACS of 4 using the VNC data. Forty-eight of the remaining 72 patients were male (66.7 %). The mean age was 61.3 ± 10.8 years (IQR: 54.4–69.0 years; range: 36–85 years).

For non-contrast-enhanced scans, computed tomography dose index (CTDI) was 4.15 ± 0.29 and dose length product (DLP) was 64.32 ± 9.43 resulting in an estimated effective dose of 1.15 ± 0.17 mSv. For contrast-enhanced scans, CTDI was 17.01 ± 2.90 and DLP was 266.6 ± 60.82 resulting in a total effective dose of 4.81 ± 1.09 mSv. The average time between non-contrast and contrast-enhanced scans was 4.63 min.

### Comparison between calcium scoring from true and virtual non-contrast images

**Fig. 1:**
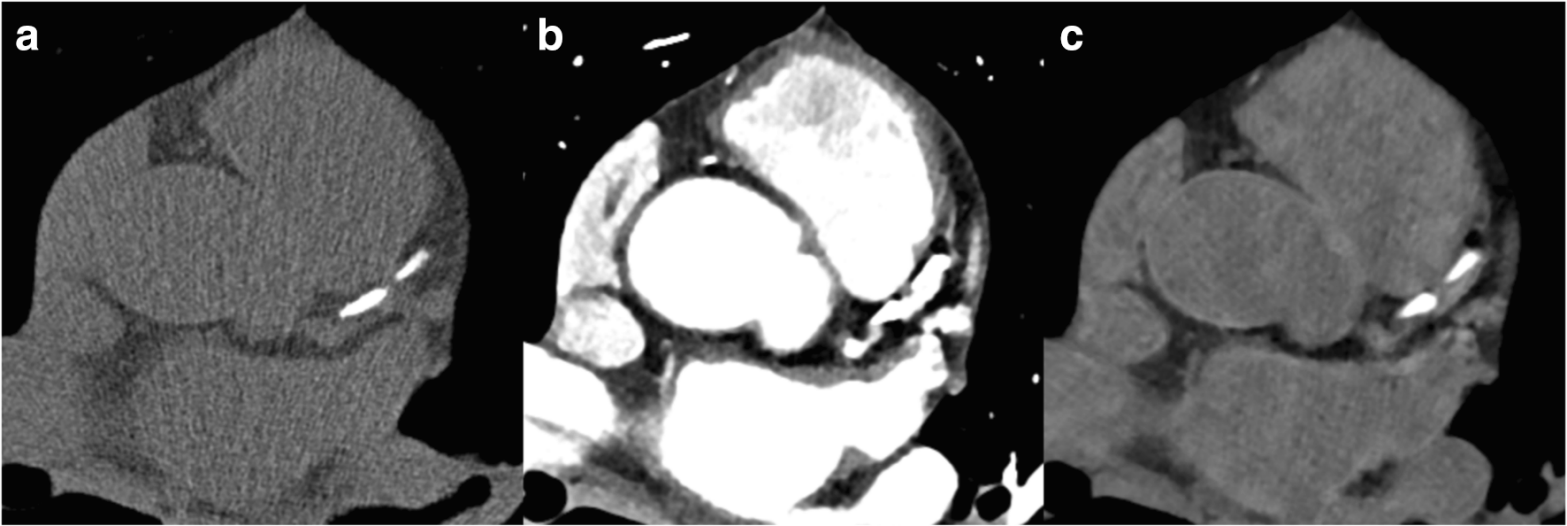
True non-contrast image from a native scan (**a**), contrast-enhanced image (**b**), and virtual non-contrast image (**c**) from a coronary computed tomography angiography (coronary CTA)

The use of TNC resulted in a mean calcium score of 178 with a minimum value of 1, a maximum value of 2048, and an inter-quartile range of 22–282. Calculations of calcium scoring using VNC spectral images of the same patients resulted in a mean calcium score of 43 with a minimum value of 0, a maximum value of 518, and an inter-quartile range of 7–56. Only one patient with a CACS of 7 on TNC images was wrongly classified as having a CACS of 0 on VNC images. Figure 1 shows a representative example of TNC, contrast-enhanced, and VNC images of a patient.

**Fig. 2:**
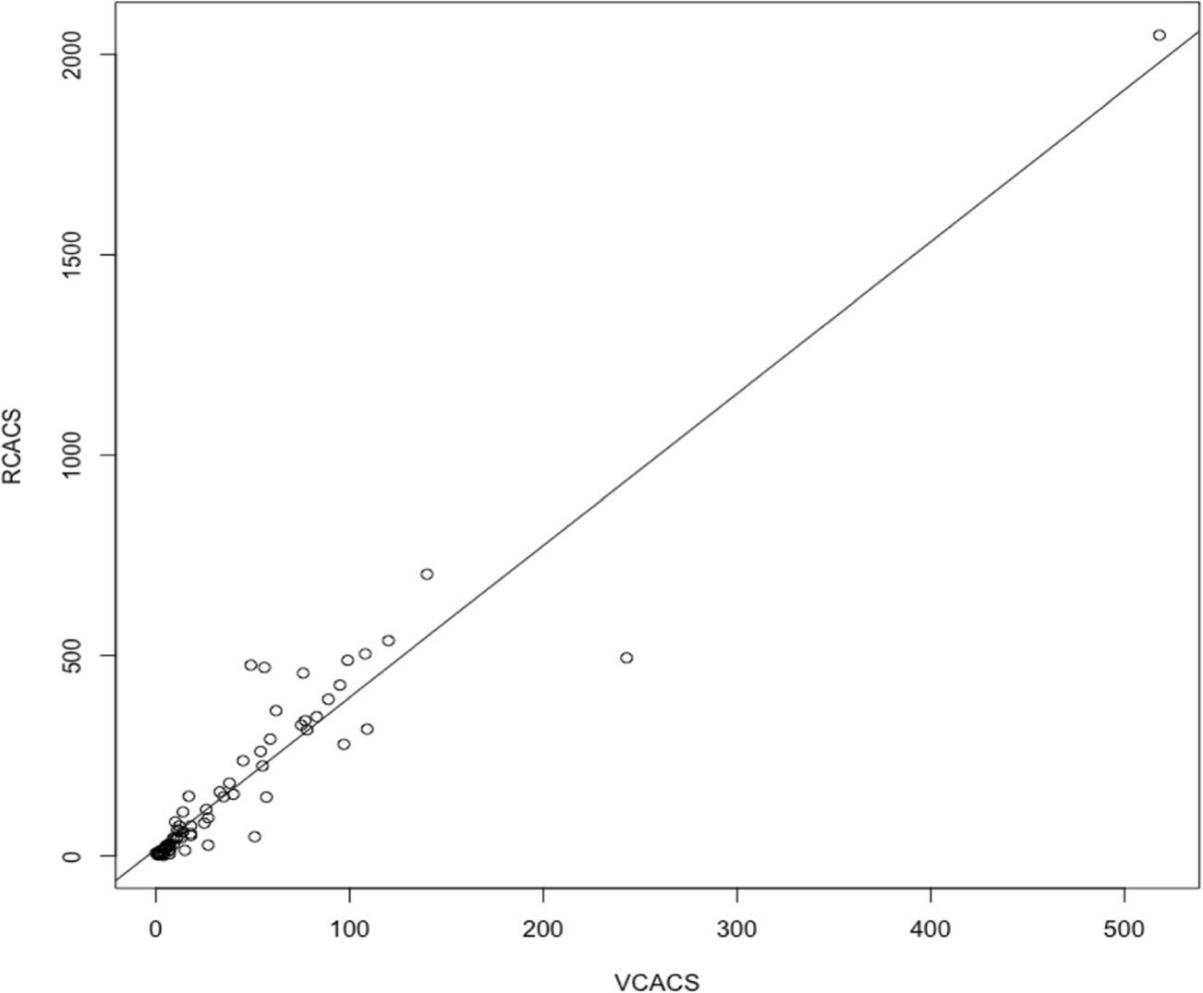
Correlation between calcium scoring using true and virtual non-contrast images. RCACS, calcium scoring using true non-contrast images; VCACS, calcium scoring using virtual non-contrast images

There was a very high and significant correlation of calcium scoring calculated from TNC and VNC images (0.95) (*p* < 0.001) and an acquired power of 100 % for this result. Overall difference of means between methods was 4.14-fold. For the determination of exact proportionality factor, linear regression using the method of least squares was applied (Fig. 2). The slope was 3.83 indicating an underestimation of calcium scores obtained using the VNC data compared to those obtained from the TNC data by that factor. The intercept was 3.8 indicating a correct association between methods.

**Fig. 3:**
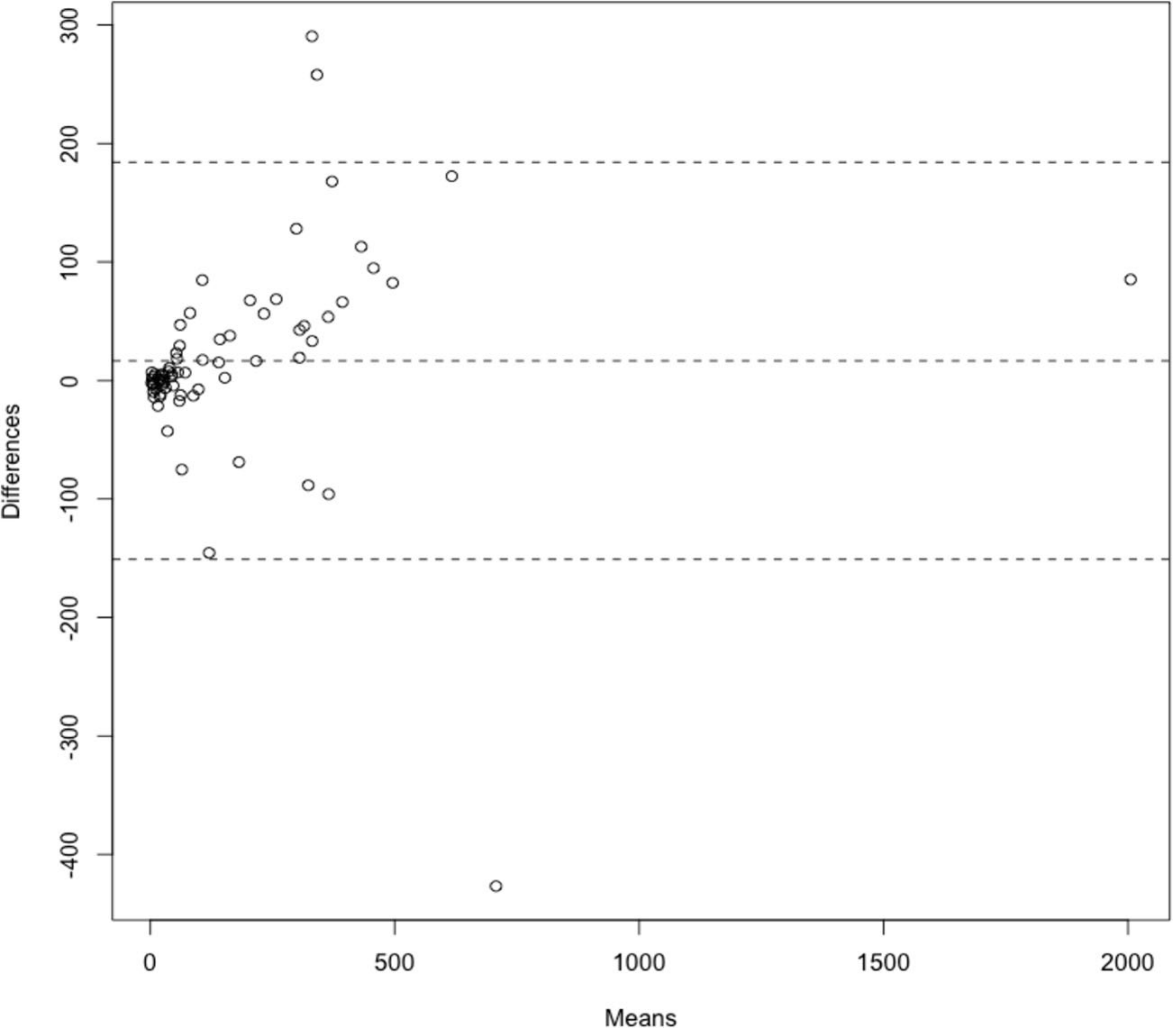
The Bland-Altman plot for the ratio between calcium scoring using true and virtual non-contrast images

Using the slope derived from the linear regression model as proportionality constant, visual analysis of the Bland-Altman plot was performed. When the results from the VNC data are multiplied by this slope, CACS shows good accordance between both methods as shown in Fig. 3. Only 3 of 72 cases (4.2%) are located outside the ± 1.96 standard deviation range of difference. Regarding the grading of coronary artery disease (low risk: CACS = 1–100, moderate risk: CACS = 101–400, high risk: CACS > 400), 60 patients (83.3 %) were assigned to the same category, 4 in the category above, and 8 in the category below using VNC data as compared to TNC data.

## Discussion

The determination of the coronary artery calcium score is an essential prognostic factor in patients with symptoms of coronary artery disease. Therefore, it is common practice to perform non-contrast-enhanced, electrocardiogram-gated scans for calcium quantification prior to CT angiography [17, 18]. Finding an approach for the determination of calcium score from contrast-enhanced images and thus omitting native scans preceding CT angiography would be useful for radiation dose reduction and shortening of the duration of the overall exam.

In this study including 103 patients with a correlation coefficient of 0.95, we showed that there is a very high correlation between results of calcium scoring from real non-contrast images as currently performed in clinical routine and VNC images derived from contrast-enhanced imaging of a 64-slice single-source dual-layer spectral CT system through a software package certified for medical use and commercially available.

These results in general are in line with previous studies which also have shown good results of calculation of calcium score from VNC images. Schwarz et al demonstrated a correlation coefficient of 0.95 in 36 patients in a dual-source CT system and Fuchs et al showed a high agreement between methods with a correlation coefficient of 0.96 in 52 patients using a rapid kVp-switching system and applying a reduced dose of contrast media [8, 9]. Nevertheless, this is the first study to evaluate the accuracy of CACS from VNC imaging computed from spectral data on a SDCT scanner in comparison to standard non-contrast imaging in a more representative patient cohort with clinically approved software.

Additionally, it is of note that contrary to other studies, patients with a CACS of zero were excluded in our study to better understand the correlation of the measured values [8, 9]. However, the intercept was 3.8 and therefore close to zero, indicating the correct association between the methods. Furthermore, of the 31 patients with CACS of zero, none exhibited virtual CACS of ≥ 5 indicating a low rate of false-positive results. The false-positive result was visually assessed and seems to be most likely caused by reconstruction artefacts as in very few occasions wall adherent contrast agent was not fully subtracted from the image.

Despite the exclusion of patients with a calcium score of zero, Pearson’s correlation coefficient of 0.95 still indicates one of the highest correlations of calcium scoring values between true and virtual non-contrast images and 83.3% of patients were classified in the same category comparing CACS calculated from TNC and VNC [8, 9, 19, 20].

Nevertheless, there is still a small residual discrepancy with 16.7% of patients being classified in a higher or lower risk category when using VNC as compared to TNC. This discrepancy does not necessarily need to be attributed to the difference in methods as a previous study showed a notable inter-scan variability within 5 min using the same CT equipment [21]. Additionally, different standard kernels used and processing through dedicated software might further influence the outcome of CACS [22].

For proper transfer of results between methods, a proportionality factor needs to be applied as analysis of real non-contrast images showed a calcium score 3.83 times as high as calcium scores analyzed in VNC images. As virtual non-contrast is based on decomposition into two different materials (soft tissue and iodine), attenuation of calcium can be reduced as a consequence. In a study published recently, Nadjiri et al showed that there might be two reasons for the underestimation of calcium scoring in VNC data: a slight underestimation of the plaque volume and, more importantly, an underestimation of plaque attenuation in VNC images; a reduced attenuation of Ca-plaques is to expect in the VNC reconstruction. Therefore, certain plaques will be excluded from the semiautomatic analysis as they will not exceed the threshold of 130 HU in the VNC reconstruction [5].

The goal of this study was to show a way of reducing radiation in analysis of CACS using a dual-layer SDCT. Tube-based dual-energy CT systems have been reported to increase radiation exposure depending on the patient’s heart rate, protocol, and scanner generation [23, 24]. Nevertheless, improved conventional detectors with higher dose efficiency as well as the omission of native scans reduced overall radiation exposure compared to current clinical standard procedures in CT-based evaluation of coronary artery disease by approximately 20–25% [25, 26]. When omitting the native scan in this study, effective dose could be reduced by 19.3%. The effective dose of 4.81 mSv for the contrast-enhanced scan with our scanner is comparable to radiation doses commonly published in the literature for this type of examination, although lower effective doses can be achieved when using e.g. 320-detector-row single-source CT scanners or dual-source CT scanners [27, 28].

Despite its clinical setting, high number of subjects, and good correlation, our study has several limitations. First of all, CT-based true non-contrast imaging was used as a reference standard. Although beam hardening and blooming have been described in the context of calcified coronary artery lesions and could lead to artifacts, they could also occur in native scans [8, 29]. Nevertheless, there is no other feasible method available in a clinical setting.

There are some differences in our results from this study compared to previously published findings, where Nadjiri et al reported a proportionality factor of 1.83. Some of these differences could be attributed to difference in sample size, exclusion of patients with CACS of zero, and different software and conversion tool used. Additionally, Nadjiri et al used a threshold of 90 HU for CACS whereas, in this study, the threshold was set to 130 HU as commonly used for Agatston scoring [5, 7]. For transfer into daily clinical use, confirmation of results in a multi-center study and precise definition of extent and causes of proportionality factor is needed.

To conclude, this is the first study to demonstrate that the determination of coronary artery calcium score is feasible using VNC images obtained from a dual-layer spectral detector CT in a representative patient cohort. By applying a correction factor, the results show good agreement with the standard technique. This could obviate the need for native scans typically used for CACS, thereby facilitating radiation dose reductions. Nevertheless, translation into clinical practice is subject to further studies and evaluation of causes of needed proportionality factor.

## Abbreviations

CACS: Coronary artery calcium scoring
CAD: Coronary artery disease
CT: Computed tomography
CTA: Computed tomography angiography
CTDI: Computed tomography dose index
DLP: Dose length product
HU: Hounsfield units
SDCT: Spectral detector CT
TNC: True non-contrast
VNC: Virtual non-contrast
